# Preferential Genetic Pathways Lead to Relapses in Adult B-Cell Acute Lymphoblastic Leukemia

**DOI:** 10.3390/cancers16244200

**Published:** 2024-12-17

**Authors:** Josgrey Navas-Acosta, Alberto Hernández-Sánchez, Teresa González, Ángela Villaverde Ramiro, Sandra Santos, Cristina Miguel, Jordi Ribera, Isabel Granada, Mireia Morgades, Ricardo Sánchez, Esperanza Such, Susana Barrena, Juana Ciudad, Julio Dávila, Natalia de Las Heras, Alfonso García-de Coca, Jorge Labrador, José Antonio Queizán, Sandra Martín, Alberto Orfao, Josep-María Ribera, Rocío Benito, Jesús María Hernández-Rivas

**Affiliations:** 1 IBSAL, IBMCC, CSIC, Centro de Investigación del Cáncer, University of Salamanca, 37007 Salamanca, Spain; idu036033@usal.es (J.N.-A.); alhesa@usal.es (A.H.-S.); tergonma@usal.es (T.G.); angelavr@usal.es (Á.V.R.); sandrasantos@usal.es (S.S.); cristinamiga@usal.es (C.M.); beniroc@usal.es (R.B.); 2 Department of Hematology, Complejo Asistencial Universitario de Salamanca, 37007 Salamanca, Spain; 3 ICO-Hospital Germans Trias i Pujol, Institut de Recerca Contra la Leucèmia Josep Carreras (IJC), 08916 Badalona, Spain; jribera@carrerasresearch.org (J.R.); igranada@iconcologia.net (I.G.); mmorgades@iconcologia.net (M.M.); jribera@iconcologia.net (J.-M.R.); 4 Department of Hematology, Hospital Doce de Octubre Hospital, 28041 Madrid, Spain; ricard.sanchez@salud.madrid.org; 5 Department of Hematology, Hospital Universitari i Politecnic La Fe, 46026 Valencia, Spain; such_esp@gva.es; 6 Department of Cytometry, University of Salamanca, 37007 Salamanca, Spain; subadelfa@usal.es (S.B.); ciudad@usal.es (J.C.); orfao@usal.es (A.O.); 7 Service of Hematology, Hospital Nuestra Señora de Sonsoles, 05004 Ávila, Spain; jdavila@saludcastillayleon.es; 8 Service of Hematology, Hospital Universitario de León, 24071 León, Spain; nherasr@saludcastillayleon.es; 9 Department of Hematology, Hospital Clínico de Valladolid, 47003 Valladolid, Spain; agarciaco@saludcastillayleon.es; 10 Department of Hematology, Hospital Universitario Burgos, 09006 Burgos, Spain; jlabradorg@saludcastillayleon.es; 11 Department of Hematology, Hospital General de Segovia, 40002 Segovia, Spain; jqueizan@saludcastillayleon.es; 12 Molecular Biology Unit, Hospital Regional Universitario de Málaga, 29010 Málaga, Spain; sandra.martin.tellez.sspa@juntadeandalucia.es; 13 Department of Medicine, University of Salamanca, 37007 Salamanca, Spain

**Keywords:** B-cell ALL, relapse, NGS, mutational dynamics, *IZKF1* ^plus^, *RAS* mutations, *TP53*

## Abstract

Adult B-cell acute lymphoblastic leukemia (B-ALL) is characterized by genetic heterogeneity and a high relapse rate, affecting over 40% of adults. However, the mechanisms leading to relapse in adults are poorly understood. Forty-four adult B-ALL patients were studied at both diagnosis and relapse by next-generation sequencing (NGS). Four main genetic pathways leading to relapse in adults were identified: *IKZF1*^plus^ genetic profile, *RAS* mutations and *TP53* alterations in Ph-negative B-ALL and acquisition of *ABL1* mutations in Ph-positive patients. The most frequently deleted gene at diagnosis was *IKZF1* (52%), and 70% of these patients had *IKZF1*^plus^ profile. Notably, 88% of patients with *IKZF1*^plus^ at diagnosis retained this genetic profile at relapse. Conversely, the acquisition of *RAS* mutations or the expansion of subclones (normalized variant allele frequency < 25%) present from diagnosis were observed in 24% of Ph-negative patients at relapse. In addition, 24% of relapses in the Ph-negative cohort could potentially be driven by *TP53* alterations. Of these cases, five presented from diagnosis, and four emerged at relapse, mostly as “double-hit” events involving both *TP53* deletion and mutation. In Ph-positive B-ALL, the main genetic finding at relapse was the acquisition of *ABL1* mutations (86%). Three clonal evolution patterns were identified: the persistent clone trajectory (25%), the expanding clone trajectory (11%) and the therapy-boosted trajectory (48%). Our results reveal the presence of preferential biological pathways leading to relapse in adult B-ALL. These findings underscore the need for personalized therapeutic strategies to improve clinical outcomes in adult patients with B-ALL.

## Simple Summary

Adult B-cell acute lymphoblastic leukemia (B-ALL) is characterized by genetic heterogeneity and high relapse rate. Forty-four adult B-ALL patients were studied at both diagnosis and relapse by next-generation sequencing (NGS) in order to identify genetic alteration driving relapse. Four main genetic pathways leading to relapse in adults were identified: *IKZF1*^plus^ genetic profile, *RAS* mutations and *TP53* alterations in Philadelphia chromosome (Ph)-negative B-ALL and acquisition of *ABL1* mutations in Ph-positive patients. In addition, three clonal evolution patterns were identified: the persistent clone trajectory (25%), the expanding clone trajectory (11%) and the therapy-boosted trajectory (48%). Our results reveal the presence of preferential biological pathways leading to relapse in adult B-ALL.

## 1. Introduction

B-cell acute lymphoblastic leukemia (B-ALL) is a hematologic neoplasm characterized by a high level of genetic heterogeneity [1]. Despite significant advances in the development of therapeutic schemes based on risk stratification that have led to a high cure rate in pediatric patients [2], the landscape in the adult population continues to be unfavorable. Over 40% of adults relapse, and only 20–40% achieve a second complete remission with five-year post-relapse survival rates less than 10% [3]. Therefore, relapsed disease represents one of the main challenges in the clinical management of adult B-ALL.

At relapse, patients often develop increased resistance to therapy mainly due to the presence of selected or treatment-induced genetic alterations [4,5]. In recent years, next-generation sequencing (NGS) has provided valuable information to characterize the evolutionary processes of B-ALL and to identify somatic events that confer selective advantages during treatment. These events include mutations typically emerging during therapy in genes such as glucocorticoid receptors (*NR3C1*), *CREBBP* and *WHSC1* (modifying the response to glucocorticoids), *NT5C2* and *MSH6* (modulating the response to thiopurines), *ABL1* (affecting the response to tyrosine kinase inhibitors) and others [6,7,8].

Recent research has shown that genomic abnormalities in *IKZF1* and *TP53* genes could be prognostic biomarkers of relapse and improve current risk stratification algorithms at diagnosis [9,10,11,12]. The *IKZF1*^plus^ genetic profile has been recently defined, which is characterized by the presence of *IKZF1* deletions in combination with at least one deletion in *CDKN2A/B*, *PAX5* or *PAR1*, in the absence of *ERG* deletions [13]. This profile has been associated with worse prognosis compared to single *IKZF1* deletions. However, most studies have focused on pediatric patients [14,15,16,17], and data in adult patients are still limited [18,19,20,21,22]. In addition, it has been recently proposed that adult ALL patients with mutations in genes associated with myeloid neoplasms have an adverse clinical outcome [23]. However, to date, few studies have evaluated the prognosis of these mutations in this group of patients [23,24,25,26].

Therefore, the mechanisms leading to relapse of B-ALL are not completely understood in adult patients. The aim of this study was to identify genetic alterations driving relapse in adult B-ALL from paired samples (diagnosis–relapse), using a customized NGS panel.

## 2. Materials and Methods

### 2.1. Patients and Samples

For this study, 44 adult B-ALL patients (median age: 42 years, range: 18–72 years), who relapsed after achieving complete remission, were included. Adult patients were treated according to PETHEMA protocols (NCT00494897, NCT02036489, NCT00853008, NCT01366898, NCT01540812, NCT01376427, NCT01491763 and NCT04179929). In total, 91 bone marrow samples were analyzed. Forty-one patients had paired diagnosis–relapse samples while 3 patients had samples from three evolutionary moments (diagnosis-first relapse-second relapse). Samples with <10% of blast infiltration were sorted by flow cytometry, and only purified blasts were used to perform NGS study to ensure adequate sensitivity (Supplementary Materials Table S1). The main characteristics of adult B-ALL patients are shown in Table 1 and Supplementary Materials Table S2.

The study was approved by the Clinical Research Ethics Committee of the Hospital Universitario de Salamanca. Participants provided written informed consent before entering the study.

### 2.2. Fluorescence In Situ Hybridization (FISH)

Interphase FISH was performed on peripheral blood or bone marrow samples using the following commercial probes: *BCR::ABL1*, *ETV6::RUNX1*, *TCF3::PBX1*, *KMT2A* (*MLL*), *CRLF2*, *ABL1*, *ABL2, MYC*, *BLC2* and *TP53* (Vysis, Abbott Laboratories, Chicago, IL, USA) (Supplementary Materials Table S3).

### 2.3. Next-Generation Sequencing (NGS)

NGS was performed following the hybridization capture targeted sequencing methodology, using an updated version of the custom NGS panel designed and validated in our laboratory [27]. We have analyzed the presence of (1) Somatic mutations (single nucleotide variants [SNVs] or small insertions/deletions [INDELs]) in 150 genes involved in the evolution of B-ALL; (2) Most recurrent gene fusions (*ETV6::RUNX1*, *TCF3::PBX1*, *BCR::ABL1*, *KMT2A* rearrangement (*KMT2A*r) and *CRLF2* rearrangement (*CRLF2r*)); (3) Aneuploidies (gains and losses) in chromosomes 4, 7, 8, 9, 10, 17 and 21; (4) Copy number variations (CNVs) in *IKZF1*, *CDKN2A/B*, *PAX5*, *BTG1*, *RB1*, *ETV6*, *EBF1*, *TP53* and *VPREB1/2*. The libraries were sequenced on an Illumina NextSeq^TM^ 500 platform using the NextSeq 500/550 Mid Output Kit v2.5 (300 Cycles) (Illumina Inc., San Diego, CA, USA). Median coverage of target regions was 3 million reads/sample. Approximately 90% of the bases (of all the regions included in the design) had a depth of coverage of >100×.

### 2.4. Clonal Evolution and Pathway Analysis

Variant allele frequency (VAF) was used as a quantitative measure to determine the proportion of leukemic cells with specific genetic variants. For this, we normalized VAF in relation to the number of blasts at each time point. A normalized VAF of <25% was considered subclonal [28,29,30,31].

Types of relapses based on timing after diagnosis are classified as: very early relapse that occurs within 18 months of diagnosis; early relapse, more than 18 months after initial diagnosis but less than 6 months after discontinuation of first-line treatment; and late relapse, more than 6 months after discontinuation of first-line treatment.

Low hypodiploid (LH) patients were excluded for *IKZF1*^plus^ genetic profile analysis due to the high frequency of loss of chromosomes 7 and 9.

### 2.5. Statistical Analysis

Differences between diagnosis and relapse were compared by paired-samples t Student or Wilcoxon tests, as appropriate (determined by the Kolmogorov–Smirnov test to verify normality). *p* values < 0.05 were considered statistically significant. Analyses and graphical representations were performed using GraphPad Prism 8.2.1 (GraphPad Software, San Diego, CA, USA) and R 4.0.2 (R Foundation for statistical Computing, Vienna, Austria), using ComplexHeatmap, fishplot, circlize and tidyverse packages.

## 3. Results

### 3.1. Genetic Landscape of Adult Relapsed B-ALL

Genomic analysis by NGS revealed that all adult B-ALL patients had at least one genetic alteration (single nucleotide variant (SNV), small insertion/deletion (INDEL), copy number variation (CNV), aneuploidy and/or gene fusion) at diagnosis and/or relapse. Eighty-one distinct genetic alterations were identified in 44 patients. A total of 44 somatic mutations (SNV/INDELS) were detected, including 32 missense (73%), eight frameshift (18%), three non-frameshift (7%) and one splicing (2%) mutations in 20 genes, of which nine genes were recurrently mutated in this cohort of patients (identified in more than two patients) (Supplementary Materials Table S4). In addition, 12 CNVs (involving nine genes and three deleted regions), and complete gains and/or losses of 18 chromosomes were detected (Supplementary Materials Table S5), as well as six rearrangements (*BCR::ABL1*, *TCF3::PBX1*, *KMT2Ar*, *CRLF2r*, *MYCr* and *BCL2r*) (Supplementary Materials Table S3).

**Figure 1:**
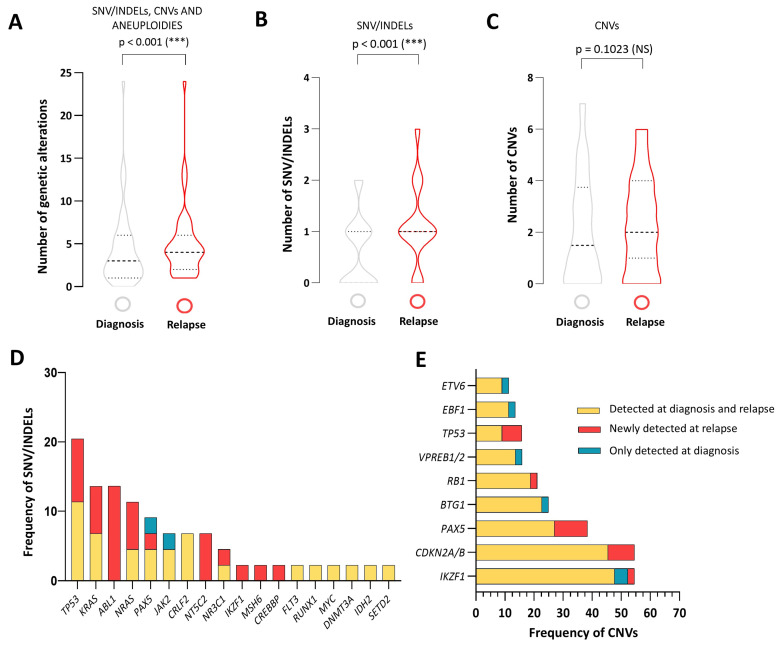
Genetic landscape of adult relapsed B-ALL. Violin plot showing the differences in the number of (**A**) all genetic alterations (SNV/INDELs, CNVs and aneuploidies) (**B**) SNV/INDELs and (**C**) CNVs between diagnosis and relapse in adult B-ALL. Frequency of (**D**) SNV/INDELs and (**E**) CNVs (deletions) in genes regulating B-cell development and cell cycle at both diagnosis and relapse in adult B-ALL patients. The yellow color means present at diagnosis and relapse, the blue color means lost at relapse and the red color means acquired at relapse. *TP53* deletion was associated with loss of chromosome 17 or 17p deletion. NS: not significant; *** *p* < 0.001. CNV: Copy number variation. SNV/INDELs: single nucleotide variant/small insertions/deletions.

A total of 27 mutations were identified at diagnosis in adult patients. At relapse, 28 new mutations were found, and two of mutations present at diagnosis were lost. A significant increase in the number of genetic alterations in adult B-ALL patients at relapse compared to diagnosis was identified (5.11 vs. 4.4, *p* < 0.001) (Figure 1A), indicating the presence of a more complex mutational profile at relapse. When the analysis was performed according to each type of genetic alteration, the number of SNV/INDELs showed significant differences between diagnosis and relapse (0.6 vs. 1.14, *p* ≤ 0.001) (Figure 1B). However, we could not find differences in the number of CNVs at both diagnosis and relapse (2.11 vs. 2.30, *p* = 0.1023) (Figure 1C).

The most frequently observed mutations at both diagnosis and relapse were *TP53* (21%), *KRAS* (13.6%), *NRAS* (9%) and *CRLF2* (7%) mutations (Figure 1D). In contrast, mutations detected exclusively at relapse were frequently found in drug resistance-related genes in adult B-ALL (11/44, 25%): *ABL1* mutations (T315I, Y253H and F359V) predominated in relapsed Philadelphia chromosome (Ph)-positive patients (6/7, 86%), while *NT5C2* (R238L, R39Q and R667Q), *NR3C1* (R714L and Q74fs), *MSH6* (T1085fs) and *CREBBP* (S1436R) were mainly identified in relapsed Ph-negative patients (5/37, 14%). (Figure 1D).

Moreover, gene deletions were the most common CNVs, mainly affecting genes related to B-cell development and cell cycle control. In most cases, they were present at diagnosis and maintained at relapse, whereas the presence of new deletions at relapse was rare: *IKZF1* (48% at diagnosis and relapse vs. 2% exclusively at relapse), *CDKN2A/B* (46% vs. 9%), *PAX5* (27% vs. 11%), *BTG1* (23% vs. 0%), *RB1* (19% vs. 2.3%), *VPREB1/2* (14% vs. 0%), *EBF1* (11% vs. 0%) and *ETV6* (9% vs. 0%). Loss of chromosome 17 or 17p deletion (*TP53*) was the most frequent chromosomal region alteration at relapse (9% at diagnosis and relapse vs. 7% exclusively at relapse) (Figure 1E).

### 3.2. Preferential Genetic Pathways Leading to Relapse in Adult B-ALL

To study the different genetic pathways involved at relapse in adult B-ALL patients, genetic alterations and their dynamics from diagnosis to relapse were analyzed. Four main genetic pathways were identified that could explain 82% of relapses in adult B-ALL: *IKZF1*^plus^ genetic profile, *RAS* mutations and *TP53* alterations in Ph-negative B-ALL while *ABL1* mutations were acquired at relapse in Ph-positive patients.

**Figure 2:**
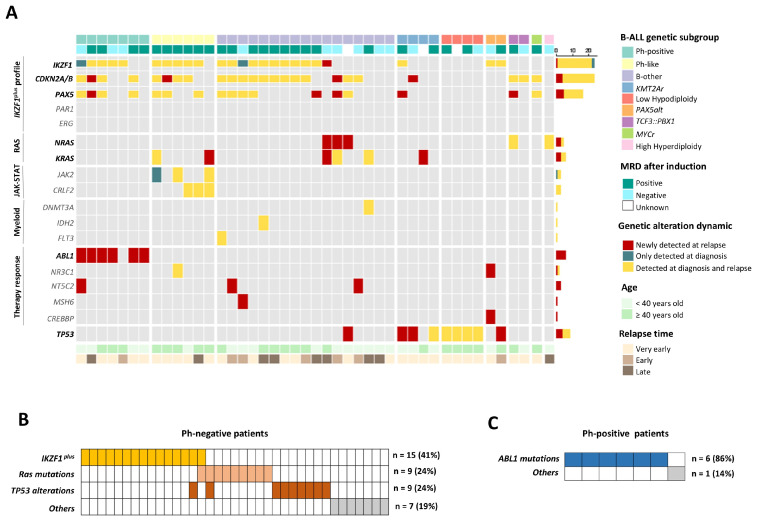
Mutational dynamics in progression to relapse in adult B-ALL. (**A**) Comprehensive landscape of genetic alterations in adult relapsed B-ALL grouped into biological pathways and the *IKZF1*^plus^ profile. Genes are ordered in rows, and each column represents one patient. The color scale of the genetic alteration indicates their dynamics at relapse. The yellow color indicates that the genetic alteration dynamic remains from diagnosis to relapse; the blue color, disappears at relapse and the red, appears only at relapse. In the *IKZF1*^plus^ block, only CNV dynamic is represented by rectangles. The mutation dynamic is represented in the rest of the figure by square. (**B**) Genetic pathways that lead to relapse in adult Ph-negative B-ALL patients: *IKZF1*^plus^, *RAS* mutations and *TP53* alterations. (**C**) Genetic pathways that lead to relapse in adult Ph-positive B-ALL patients: *ABL1* mutations. Each column corresponds to a patient, and the presence of genetic alterations is grouped according to type and shown in colors. MRD: Minimal/measurable residual disease. *TP53* alteration: *TP53* Mutations with/without deletions.

A high frequency of *IKZF1* deletions was observed in adult relapsed B-ALL, often co-occurring with *CDKN2A/B* and *PAX5* deletions (Figure 2A) (Supplementary Materials Figure S1A). Among the 23 patients with *IKZF1* deletions at diagnosis, 70% (16/23) met the *IKZF1*^plus^ genetic profile definition. The *IKZF1*^plus^ profile was associated with positive minimal/measurable residual disease (MRD) after induction compared to the rest of the relapsed patients in our cohort (88% vs. 52% *p* = 0.0194). Moreover, 88% of patients with *IKZF1*^plus^ at diagnosis retained this genetic profile at relapse. We observed that out of the seven patients with *IKZF1* deletions who did not meet the *IKZF1*^plus^ definition at diagnosis, three acquired this genetic profile at relapse, with the acquisition of *PAX5* and/or *CDKN2A/B* deletions (Supplementary Materials Figure S1B). *IKZF1^plus^* was predominantly found in Ph-negative adult patients at relapse (15/37, 41%) (Figure 2B). Most Ph-like patients had the *IKZF1^plus^* profile at diagnosis (5/6), which persisted at relapse. Notably, these Ph-like patients did not acquire additional alterations at relapse (Figure 2A).

**Figure 3:**
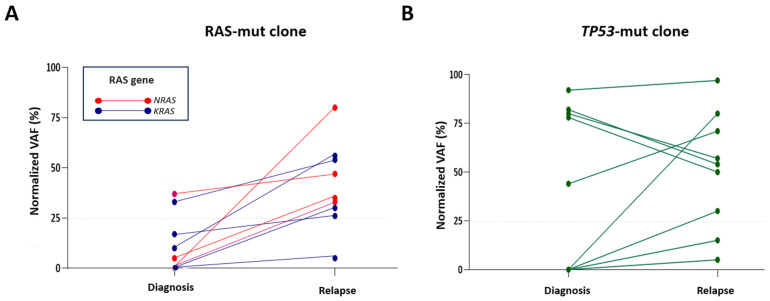
Dynamics of *RAS* and *TP53* mutations during evolution to relapse. Normalized VAF at both diagnosis and relapse of mutations detected in (**A**) *NRAS* and *KRAS* and (**B**) *TP53* genes in adult B-ALL patients. VAF: Variant allele frequency.

*RAS* mutations (*KRAS* and/or *NRAS*) were identified in 14% (5/37) of the Ph-negative patients at diagnosis, with 60% of these mutations being subclonal (normalized VAF < 25%). At relapse, all pre-existing subclones expanded, becoming the main clone (normalized VAF ≥ 25%), and four patients acquired *RAS* mutations that were not present at diagnosis, increasing the number of Ph-negative patients with *RAS* mutations at relapse to 24%. In 88% (8/9) of cases, *RAS* mutations were found in the predominant clone at relapse (Figure 3A), with mutation at G12 amino acid residues being the most common (78%) (Supplementary Materials Table S4). There was a single case (P1) that acquired a subclonal *KRAS* mutation at first relapse, which expanded at second relapse (Supplementary Materials Table S4). Interestingly, only one patient (P27) had both the *RAS* mutations and *IKZF1^plus^* profile at relapse (Figure 2B). In this patient, a *KRAS* mutation was detected in a subclone at diagnosis, suggesting that these alterations could be present in different clones (Supplementary Materials Figure S2).

**Figure 4:**
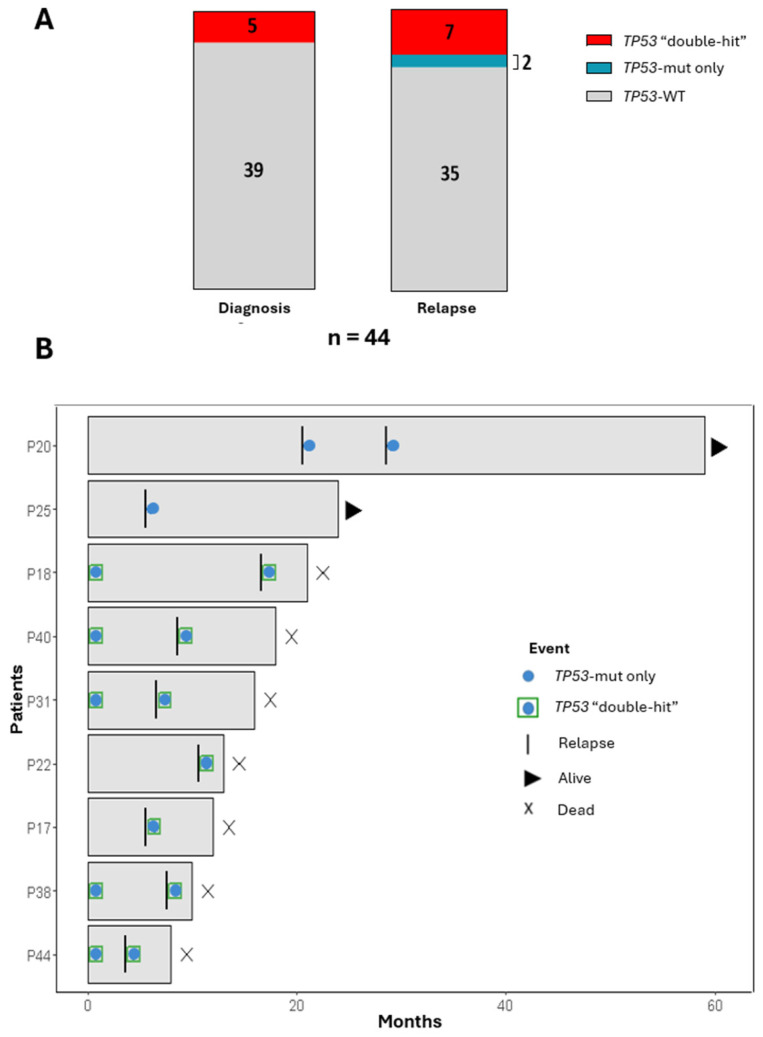
Characterization of *TP53* status during the disease progression (**A**) Histogram representing the frequencies of *TP53* alterations across diagnosis and relapse (**B**) Swimmer plot representing how a *TP53* molecular status changes across disease phases (diagnosis and relapse). Each horizontal bar represents a patient and shows the evolution of the *TP53* status from diagnosis to relapse: WT (empty), only mutated (blue circle) and double-hit: mutation and deletion (blue circle with green square). The current status of the patient (alive or dead) is also indicated. WT: Wild type.

*TP53* alterations were found in 5 of the 37 Ph-negative patients (14%) at diagnosis, all presenting a “double-hit” (deletion and mutation) in the predominant clone (Figure 3B). Analysis of relapses showed that these patients retained double-hit events, while four additional patients acquired new *TP53* alterations, either as biallelic alterations or *TP53* mutations only (Figure 4).

The evolutionary patterns of *TP53* alterations within the different B-ALL genetic subgroups were analyzed. As expected, all LH patients exhibited *TP53* double-hit at both diagnosis and relapse. In contrast, *TP53* alterations in non-LH subgroups were predominantly acquired at relapse (80%, 4/5). Interestingly, *TP53* alterations were observed in 75% of *KMT2A*r B-ALL at relapse (Figure 2A).

Of note is that we identified myeloid mutations in *FLT3* (p.D586delinsEPVHF), *IDH2* (p.R140Q) and *DNMT3A* (p.R882H) at diagnosis that were maintained at relapse in three Ph-negative patients (3/37, 8%), although they overlapped with other identified genetic pathways (Figure 2A). The mean age was 42 years (range: 34–56 years) (Supplementary Materials Table S2). Patients with *IDH2* and *DNMT3A* mutations exhibited a pro-B cells immune phenotype (CD34+, CD19+ and CD10−), while those with *IDH2* and *FLT3* mutations showed abnormal expression of myeloid antigens at diagnosis. (Supplementary Materials Table S7).

Finally, 86% of Ph-positive patients acquired *ABL1* clonal mutations at relapse. These mutations were not detected at diagnosis, even by increasing the depth of coverage to ~1000× (Figure 2A,C).

### 3.3. Adult B-ALL Patients Present Heterogeneous Patterns of Clonal Evolution During Progression to Relapse

**Figure 5:**
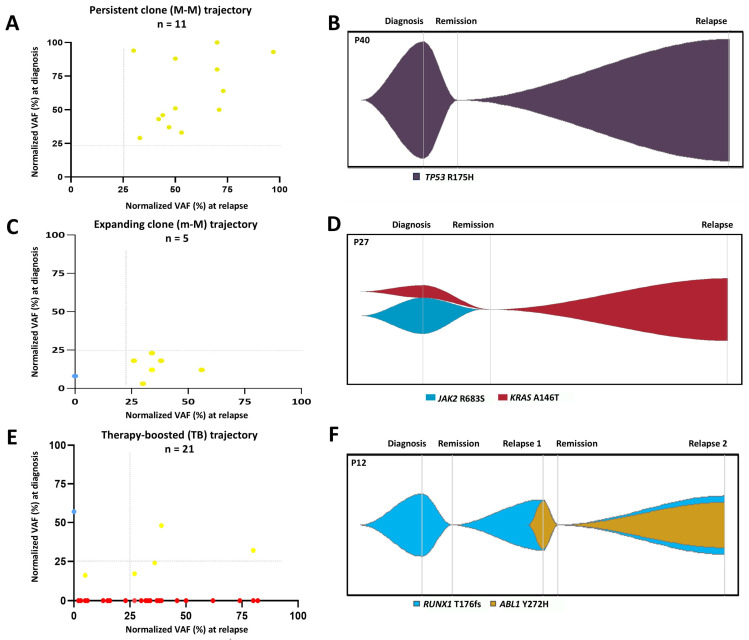
Patterns of clonal evolution in adult B-ALL. Scatter plots and illustrative representation of clonal evolution models showing. (**A**,**B**) The persistent clone (M-M) trajectory, in which the main clone (VAF ≥ 25%) present at diagnosis persists as the main clone at relapse. (**C**,**D**) The expanding clone (m-M) trajectory, where a subclone present at diagnosis expands and becomes the major clone at relapse (**E**,**F**) The therapy-boosted trajectory, in which clones at diagnosis (diagnostic clone or subclone) acquire new mutations at relapse. The yellow color means present at diagnosis and relapse, the blue color means lost at relapse and the red color means acquired at relapse. Each point represents an evolutionary moment of the patient: Diagnosis/Remission/Relapse VAF: Variant allele frequency.

To analyze mutational dynamics and establish possible cell populations, we compared the VAF of somatic mutations observed both at diagnosis and relapse. We have identified three patterns of clonal evolution:

The persistent clone (M-M) trajectory, in which the main clone (normalized VAF ≥ 25%) (11/44, 25%) present at diagnosis persists as the main clone at relapse. This trajectory was enriched in *TP53* alterations (5/11, 45%) and myeloid mutations (*FLT3*, *IDH2* and *DNMT3A* mutations) (3/11, 27%) (Figure 5A,B). This pattern is present at both very early (7/11, 64%) and late (4/11, 36%) relapses (median time to relapse: 25 months, range: 4–70 months). Notably, most of the very early relapses associated with this pattern were related to *TP53* double-hit events (4/7, 57%).

The expanding clone (m-M) trajectory (5/44, 11%), where a subclone present at diagnosis expands and becomes the major clone at relapse (normalized VAF ≥ 25%). This trajectory was observed only at very early relapses (median time to relapse: 10 months, range: 2–11 months) mainly in patients with *RAS* mutations (3/5) (Figure 5C,D).

The therapy-boosted (TB) trajectory (21/44, 48%), in which a clone at diagnosis (diagnostic clone or subclone) acquires new alterations at relapse: mostly gain of therapy resistance-associated gene mutations (*ABL1*, *NR3C1*, *NTC5C2*, *MSH6* and *CREBBP* mutations) (10/21, 48%), *RAS* mutations (5/21, 24%) and *TP53* alterations (4/21, 19%). This pattern was more prevalent at very early relapses (13/21, 62%) and early relapses (6/21, 29%) (median time to relapse: 18 months, range: 3–66 months) (Figure 5E,F).

In the remaining 16% of patients, only CNVs that were stable at both diagnosis and relapse were present.

Furthermore, at second relapses (P1, P12 and P20), we observed that the same clone responsible for the initial relapse (either the therapy-boosted, expanding or persistent clone) was the cause of the second relapse without acquiring new genetic alterations. This observation suggests that clones that show resistance to treatment from diagnosis and lead to first relapse have the ability to persist and maintain their resistance to subsequent treatments, leading to second relapses (Supplementary Materials Figure S2).

## 4. Discussion

In this study, we evaluated the presence of genetic alterations at diagnosis and relapse of B-ALL adult patients. Four main genetic pathways leading to relapse were identified: the *IKZF1*^plus^ genetic profile, *RAS* mutations and *TP53* alterations in Ph-negative patients and acquisition of *ABL1* mutations in Ph-positive patients, respectively.

We observed a significant increase in the number of genetic alterations detected at relapse. These results indicate that an increased genetic complexity could be related to the development of relapse in B-ALL. Our findings are consistent with previous studies exploring clonal evolution, particularly in pediatric cohorts [27,28,29,30,31,32].

The *IKZF1*^plus^ profile appears to be an early event in relapsed disease, as it is typically present from diagnosis. The *IKZF1*^plus^ profile is known to have an adverse prognosis in pediatric B-ALL [13,14,15,16,17,33], and an increased risk of relapse has been demonstrated especially in Ph-like patients [14,34], but research on the prognostic impact in adult B-ALL is still inconclusive [18,19,20,21,22]. In this study, the *IKZF1*^plus^ profile was present in 41% of relapsed Ph-negative adult patients, which persisted from diagnosis or was acquired in patients with single *IKZF1* deletions, suggesting that the cooperation of these genetic alterations could be contributing to the development of relapsed disease in adult patients. Furthermore, our study demonstrated that the *IKZF1*^plus^ profile correlated to MRD positivity after induction. This finding is consistent with previous studies in pediatric patients, suggesting that *IKZF1*^plus^ defines an adverse MRD-dependent prognostic profile in B-ALL [13].

Conversely, we describe a notable frequency of newly acquired *RAS* mutations at relapse, as well as the expansion of pre-existing *RAS* subclones from diagnosis. This suggests that these mutations might play a role in disease relapse. Although *RAS* mutations are prevalent at diagnosis of B-ALL, our study reinforces previous findings, indicating that their frequent occurrences in relapsed pediatric B-ALL often expanded or were acquired during treatment [30,31,35,36]. In adult B-ALL, the evidence to date is scarce, although one study demonstrated that *RAS* mutations at diagnosis negatively affected the outcomes of adult patients [37]. This is particularly relevant as these mutations may identify patients eligible for treatment with targeted inhibitors [31,36].

Strikingly, we found that *RAS* mutations and *IKZF1*^plus^ were almost mutually exclusive. Over-activation of the *RAS* and *PI3K* pathway has been demonstrated in *IKZF2* and *IKZF3* knockdown cell lines without the presence of activating mutations in these cell pathways [38]. Therefore, it is possible that *IKZF1* could work as a negative transcriptional regulator of the *RAS* pathway by binding to the *PTPN11* promoter and directly suppressing its activity, and thus tumor cells may not need both altered pathways to drive disease relapse [39].

*TP53* mutations are found in about 4–9% of adults with B-ALL at diagnosis [12,40], but this number increases up to 33% at relapse [41,42]. In fact, *TP53* alterations have been associated with a poor prognosis, a higher risk of relapse and a shorter overall survival [11,12,40,41,42,43]. Our data indicate that 24% of relapses in the Ph-negative cohort could potentially be driven by *TP53* alterations, particularly presented as biallelic events involving both deletion and mutation. These results support previous reports indicating that alteration of both *TP53* alleles is associated with an adverse prognosis in B-ALL [43], suggesting a more aggressive course.

The presence of *TP53* alterations in the LH subtype at both time points is an expected but relevant finding in this study. *TP53* mutations have been shown to represent a preleukemic event that precedes the development of 98% of LH and leads to the loss of *TP53* wild-type allele [44]. Consequently, it is likely that these alterations will persist at relapse. Moreover, we found that *TP53* alterations also occur in non-LH subgroups, although these mutations were most frequently acquired at relapse. Notably, 75% of *KMT2A*r patients in our cohort had *TP53* alterations at relapse, which have recently been associated with adverse prognosis in *KMT2A*r B-ALL [45]. In the same study, analysis of paired diagnosis–relapse samples in six *KMT2A*r patients showed an expansion of the mutant *TP53* clone or acquisition of new *TP53* alterations at relapse.

In addition, we identified myeloid mutations both at diagnosis and relapse in three Ph-negative patients, associated with abnormal expression of myeloid antigens. Mutations that are recurrent in myeloid neoplasms can also occur in adult B-ALL and correlate with advanced age and unfavorable survival outcomes [27,28,29,30]. Although the factors associated with myeloid antigens expression in patients with ALL are not fully elucidated, studies have linked these mutations with lineage infidelity [23,26]. It has been suggested that myeloid mutations may increase the risk of escape from lineage-restricted therapies and lead to relapse [25]. Therefore, while these mutations overlapped with other previously described genetic pathways, it is reasonable to consider that the combination of these alterations may have contributed to the relapse in these patients.

In Ph-positive B-ALL, a well-known mechanism of resistance is due to *ABL1* kinase domain mutations, which hinder the binding of tyrosine kinase inhibitors (TKIs). In adults, mutations in this domain have been reported in more than 80% of relapsed patients [46], although some studies suggest that these resistance mutations may be present in a relatively small leukemic subclone or arise during TKI therapy [8,46,47,48]. In our study, we confirmed previous findings, as 86% of Ph-positive patients acquired clonal mutations in *ABL1* at relapse. Resistance to imatinib caused by these kinase domain mutations can be overcome with the use of next-generation TKIs [49].

Identification of patterns of evolution has important implications for the anticipation of relapse and modulation of therapy. A highly dynamic clonal evolution was found involving different genetic lesions, showing that relapsing clones are heterogeneous and genetically more complex than those at diagnosis as described in previous studies [28,29,30,31]. Therefore, it is likely that many mechanisms converge in the selection of relapsed B-ALL clones, particularly considering the complexity of the chemotherapy protocols used in the treatment.

We have identified three patterns of clonal evolution that were previously reported [28,29,30]. Forty-eight percent of relapses in adults evolved through the TB trajectory, mediated by the appearance of new genetic alterations. The acquisition of these mutations could explain the increased resistance to treatment observed in relapsed leukemic blasts with respect to diagnosis [50]. This evolutionary pattern was mainly associated with very early relapses, suggesting a role of the therapy in the selection of mutations. These results support the model that early relapses are associated with more dynamic changes in clonal evolution [28,29].

Although our study includes, to the best of our knowledge, the largest series of adult B-ALL patients analyzed with matched samples to date, it does have limitations. Thirty-nine percent of patients in our cohort were classified as B-other, which could have been limited by the number of diagnostic tests performed in this study. Further studies should integrate complementary techniques, and larger cohorts are needed to better understand the different mechanisms of relapse within each genetic subgroup in order to guide specific therapeutic approaches.

## 5. Conclusions

In conclusion, our study reveals dramatic genetic complexity underlying relapse among adult B-ALL patients. Four main biological pathways leading to relapse were identified in adult patients: the *IKZF1*^plus^ profile, *RAS* mutations and *TP53* alterations in Ph-negative patients and the acquisition of *ABL1* mutations at relapse in Ph-positive patients. These findings underscore the need for personalized therapeutic strategies to improve clinical outcomes in adult patients with B-ALL.

## Figures and Tables

**Table 1:** Characteristics of adult B-ALL patients.

Characteristics	Adult Patients
Total *n*	44
Age at diagnosis (years), median (range)	42 (18–70)
<40 years *n* (%)	21 (48)
≥40 years *n* (%)	23 (52)
Cytogenetic classification *n* (%) *	
B-other	17 (39)
Philadelphia-positive	7 (16)
Philadelphia-like	6 (14)
*KMT2A* rearrangement	4 (9)
Low hypodiploidy	4 (9)
*TCF3::PBX1* fusion	2 (5)
*PAX5* alteration	2 (5)
*MYC* rearrangement	1 (2)
High hyperdiploidy	1 (2)
MRD after induction (%) **	
Positive (≥0.01)	26 (59)
Negative (<0.01)	14 (32)
Unknown	4 (9)
Risk group *n* (%) **	
High risk	33 (75)
Standard risk	11 (25)
Type of relapse according to time after diagnosis *n* (%) (ALL-REZ BFM classification) ***	
Very early	28 (64)
Early	7 (16)
Late	9 (20)
Time to relapse with respect to treatment	
In treatment	31 (70)
Out of treatment	13 (30)
Time from diagnosis to relapse (months), median (range)	20 (2–70)
Survival time after relapse (months), median (range)	10 (0–58)

* Minimal/measurable residual disease (MRD) at the end of induction: Positive (≥0.01% or ≥1 × 10^4^). ** Risk group stratification was designated according to PETHEMA protocols. *** Time to relapse criteria: very early, before 18 months after initial diagnosis; early, more than 18 months after initial diagnosis but less than 6 months after discontinuation of first-line treatment; and late, more than 6 months after first-line treatment.

## Data Availability

The data presented in this study are available on request from the corresponding author. The data are not publicly available due to privacy and ethical restrictions.

## Supplementary Materials

The following supporting information can be downloaded at: https://www.mdpi.com/article/10.3390/cancers16244200/s1, Table S1. Percentage of blasts and cell type sequenced at diagnosis and relapse in 44 adult patients with B-ALL. Table S2. Main characteristics of 44 adult B-ALL patients. Table S3. Fusions identified by NGS and/or FISH in 44 adult B-ALL patients. Table S4. List of all somatic mutations found by NGS in 44 adult B-ALL patients. The table includes information on change in the DNA and protein level, type of change and VAF at diagnosis and relapse. VAF: Variant allele frequency. Table S5. List of all gene deletions, CNV regions and aneuploidies found by NGS in 44 adult B-ALL patients. The table includes information at both diagnosis and relapse. Table S6. *TP53* alterations identified in adult B-ALL both at diagnosis and relapse. Table S7. Immunophenotypic markers at diagnosis of patients with myeloid gene mutations. Figure S1. Dynamics of *IKZF1* deletion in the progression to relapse in adult B-ALL. (A) Co-occurrence of *IKZF1*, *CDKN2A/B* and *PAX5* deletions at relapse in adult B-ALL. The circus plot shows associations between genetic deletions in adult patients who relapse. (B) Comparison between patients with *IKZF1* deletions at diagnosis: *IKZF1*^plus^ vs. *IKZF1*del and their association with MRD positive after induction. R: Relapse. MRD: Minimal/measurable residual disease after induction. Figure S2. Model of clonal evolution of second relapses in adult B-ALL patients. Two representative illustrations of clonal evolution from diagnosis to second relapse in adult patients with B-ALL. It is observed that the clone responsible for the first relapse is maintained or becomes the predominant clone in the second relapse. Each point represents an evolutionary moment of the patient: Diagnosis/Remission 1/Relapse 1/Remission 2/Relapse 2.
