# Supplementary material for: Preferential Genetic Pathways Lead to Relapses in Adult B-Cell Acute Lymphoblastic Leukemia
Source: Cancers (Basel). 2024 Dec 17;16(24):4200. doi: 10.3390/cancers16244200 (PMC11674736; doi:10.3390/cancers16244200)

Supplementary materials

Preferential genetic pathways lead to relapses in adult B-cell acute lymphoblastic leukemia

Authors: Josgrey Navas-Acosta[1], Alberto Hernández-Sánchez[1,2], Teresa González[1,2], Ángela Villaverde Ramiro[1], Sandra Santos[1], Cristina Miguel[1], Jordi Ribera[3], Isabel Granada[3], Mireia Morgades[3], Ricardo Sánchez[4], Esperanza Such[5], Susana Barrena[6], Juana Ciudad[6], Julio Dávila[7], Natalia de Las Heras[8], Alfonso García-de Coca[9], Jorge Labrador[10], José Antonio Queizán[11], Sandra Martín[12], Alberto Orfao[6], Josep María Ribera[3], Rocío Benito[1&], Jesús María Hernández-Rivas[1,2,13*&]

1 IBSAL, IBMCC, CSIC, Centro de Investigación del Cáncer, University of Salamanca, 37007 Salamanca, Spain; idu036033@usal.es (J.N.-A.); alhesa@usal.es (A.H.); tergonma@usal.es (T.G.); angelavr@usal.es (Á.V.); sandrasantos@usal.es (S.S.); cristinamiga@usal.es (C.M.); beniroc@usal.es (R.B.)
2 Department of Hematology, Complejo Asistencial Universitario de Salamanca, 37007 Salamanca, Spain
3 ICO-Hospital Germans Trias i Pujol, Institut de Recerca Contra la Leucèmia Josep Carreras (IJC),
08916 Badalona, Spain; jribera@carrerasresearch.org (J.R.); igranada@iconcologia.net (I.G.);
mmorgades@iconcologia.net (M.M.); jribera@iconcologia.net (J.-M.R.)
4 Department of Hematology, Hospital Doce de Octubre Hospital, 28041 Madrid, Spain; ricard.sanchez@salud.madrid.org
5 Department of Hematology, Hospital Universitari i Politecnic La Fe, 46026 Valencia, Spain; such_esp@gva.es
6 Department of Cytometry, University of Salamanca, 37007 Salamanca, Spain; subadelfa@usal.es (S.B.); ciudad@usal.es (J.C.); orfao@usal.es (A.O.)
7 Hematology Service, Hospital Nuestra Señora de Sonsoles, 05004 Ávila, Spain; jdavila@saludcastillayleon.es
8 Hematology Service, Hospital Universitario de León, 24071 León, Spain; nherasr@saludcastillayleon.es
9 Department of Hematology, Hospital Clínico de Valladolid, 47003 Valladolid, Spain; agarciaco@saludcastillayleon.es
10 Department of Hematology, Hospital Universitario Burgos, 09006 Burgos, Spain; jlabradorg@saludcastillayleon.es
11 Department of Hematology, Hospital General de Segovia, 40002 Segovia, Spain; jqueizan@saludcastillayleon.es
12 Molecular Biology Unit, Hospital Regional Universitario de Málaga, 29010 Málaga, Spain; sandra.martin.tellez.sspa@juntadeandalucia.es
13 Department of Medicine, University of Salamanca, 37007 Salamanca, Spain

* Correspondence: jmhr@usal.es
† R.B. and J.-M.H.-R. shared senior authorship.

This section contains:

- Supplementary methods.
- 7 supplementary tables.
- 2 supplementary figures.

SUPPLEMENTARY METHODS

Patients and samples

Thirty-nine percent of patients belonged to the B-other ALL group, 16% were Philadelphia chromosome (Ph)-positive, 14% were classified as Ph-like, 9% had *KMT2A* rearrangement (*KMT2A*r), 9% showed low hypodiploidy, 5% *PAX5* alteration (*PAX5*alt), 5% *TCF3::PBX1* fusion, 2% *MYC* rearrangement and the remaining 2% high hyperdiploidy. The median time from diagnosis to relapse was 20 months (range: 2-70 months), of which 64% were very early relapses within 18 months after initial diagnosis (median: 9 months, range: 2-17 months), 16% had early relapses occurring after 18 months after initial diagnosis but less than 6 months after discontinuation of first-line treatment (median: 25 months, range: 21-30 months) and 20% had late relapses appearing after 6 months after discontinuation of first-line treatment (median: 50 months, range: 38-70 months) (**Table 1) (Suppl. Table S2)**.

DNA isolation

Genomic DNA was obtained from bone marrow or peripheral blood samples. Extraction of DNA was performed using the QIAmp DNA/RNA mini kit (Qiagen, Valencia, CA, USA), following the manufacturer's recommendations. The amount of DNA was assessed by Qubit 4.0 using the Qubit™ dsDNA BR kit (Invitrogen Life Technologies, Carlsbad, CA, USA).

Analysis of sequenced samples

Variant calling and annotation for single nucleotide variants (SNVs) and small insertions/deletions (INDELs) were performed using the bioinformatic tools VarScan v2.3.9, SAMTools v1.3.1. and ANNOVAR. The databases used for variant annotation were VarSome, dbSNP, Catalogue of Somatic Mutations in Cancer (COSMIC), ClinVar, 1000 Genomes Browser and Exome Aggregation Consortium (ExAC). For copy number variations (CNVs) detection, a reference was generated with control samples sequenced with the same panel, where the normalized depth of coverage of each reference region was compared to the depth of coverage of the same region in the generated file, as previously described [1]. Also, Manta software (Illumina, San Diego, CA) was used to detect medium and large (> 50 bp) structural variants.

Validation of somatic mutations and CNVs

To validate gene deletions, the multiplex ligation-dependent probe amplification (MLPA) technique was employed using the SALSA P335 ALL-IKZF1 probemix kit (MRC Holland) according to the manufacturer's instructions. The results were interpreted with Coffalyser software (MRC Holland).

Potentially pathogenic SNV/INDELs that had either a variant allele frequency (VAF) of less than 5% or were detected only at a time point were validated using amplicon sequencing.

SUPPLEMENTARY REFERENCES

1. Montaño, A.; Hernández-Sánchez, J.; Forero-Castro, M.; Matorra-Miguel, M.; Lumbreras, E.; Miguel, C.; Santos, S.; Ramírez-Maldonado, V.; Fuster, J.L.; de Las Heras, N.; et al. Comprehensive custom NGS panel validation for the improvement of the stratification of b-acute lymphoblastic leukemia patients. *J. Pers. Med.* **2020**, *10*, 137. https://doi.org/10.3390/jpm10030137.

SUPPLEMENTARY TABLES

Table S1. Percentage of blasts and cell type sequenced at diagnosis and relapse in 44 adult patients with B-ALL.

Table S2. Main characteristics of 44 adult B-ALL patients.

Table S3. Fusions identified by NGS and/or FISH in 44 adult B-ALL patients.

Table S4. List of all somatic mutations found by NGS in 44 adult B-ALL patients. The table includes information on change at DNA and protein level, type of change and VAF at diagnosis and relapse. VAF: Variant allele frequency.

Table S5. List of all gene deletions, CNV regions and aneuploidies found by NGS in 44 adult B-ALL patients. The table includes information at both diagnosis and relapse.

Table S6. *TP53* alterations identified in adult B-ALL at both diagnosis and relapse.

Table S7. Immunophenotypic markers at diagnosis of patients with myeloid gene mutations.

SUPPLEMENTARY FIGURES

Figure S1. Dynamics of *IKZF1* deletion in the progression to relapse in adult B-ALL. A) Co-occurrence of *IKZF1, CDKN2A/B* and *PAX5* deletions at relapse in adult B-ALL. Circus plot shows associations between genetic deletions in adult patients who relapse. B) Comparison between patients with *IKZF1* deletions at diagnosis: *IKZF1*^{plus} vs. *IKZF1*del and their association with MRD positive after induction. R: Relapse. MRD: Minimal/measurable residual disease.

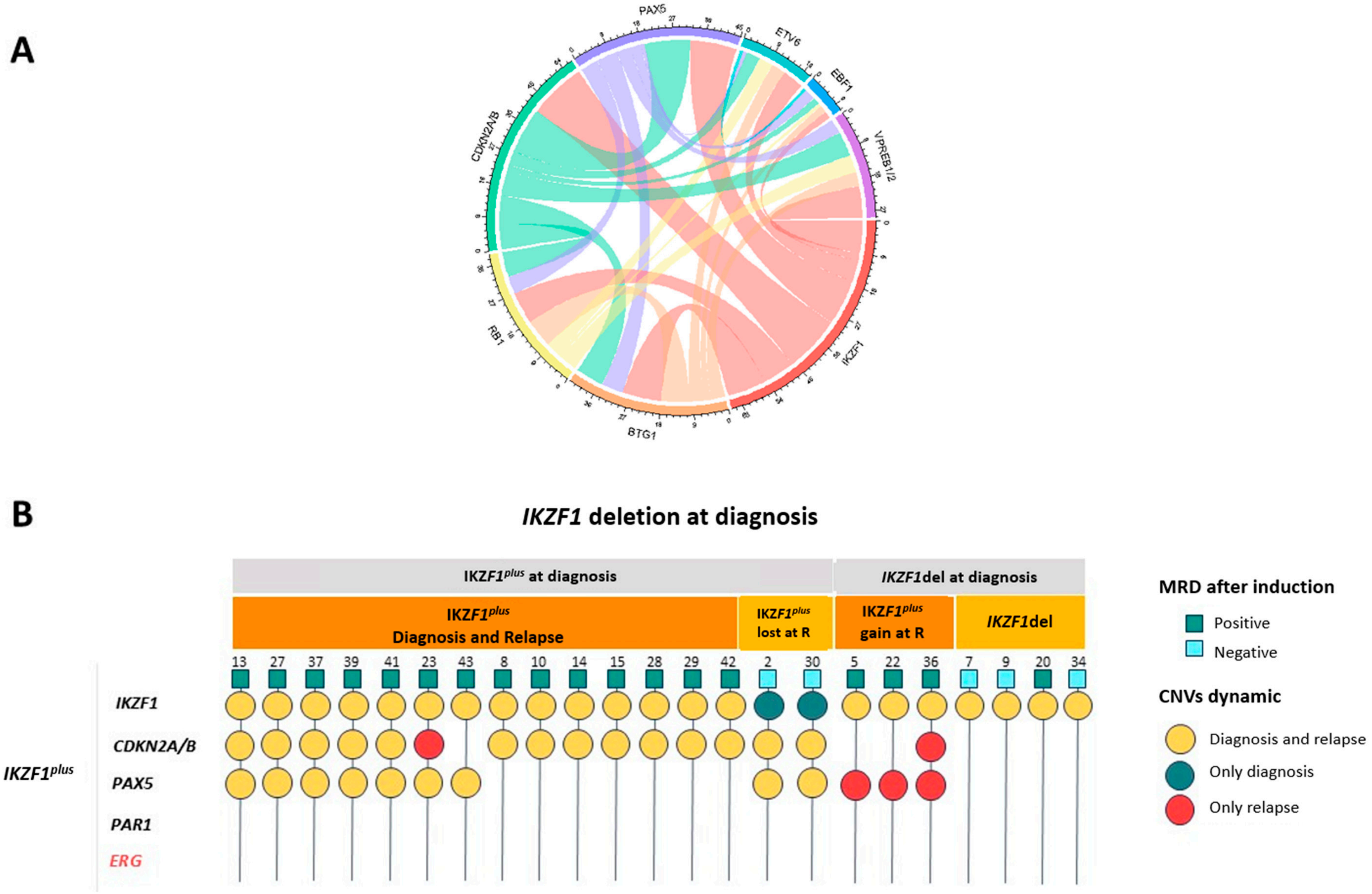
A
PAX5
ETV6
EBF1
VPREB1/2
IKZF1
BTG1
RB1
CDKN2A/B
B
IKZF1 deletion at diagnosis
IKZF1plus at diagnosis
IKZF1del at diagnosis
IKZF1plus Diagnosis and Relapse
IKZF1plus lost at R
IKZF1plus gain at R
IKZF1del
13 27 37 39 41 23 43 8 10 14 15 28 29 42 2 30 5 22 36 7 9 20 34
IKZF1
CDKN2A/B
PAX5
PAR1
ERG
IKZF1plus
MRD after induction
Positive
Negative
CNVs dynamic
Diagnosis and relapse
Only diagnosis
Only relapse

Figure S2. Model of clonal evolution of second relapses in adult B-ALL patients. Two representative illustrations of clonal evolution from diagnosis to second relapse in adult patients with B-ALL. It is observed that the clone responsible for the first relapse is maintained or becomes the predominant clone in the second relapse. Each point represents an evolutionary moment of the patient: Diagnosis/Remission 1/Relapse 1/Remission 2/Relapse 2.

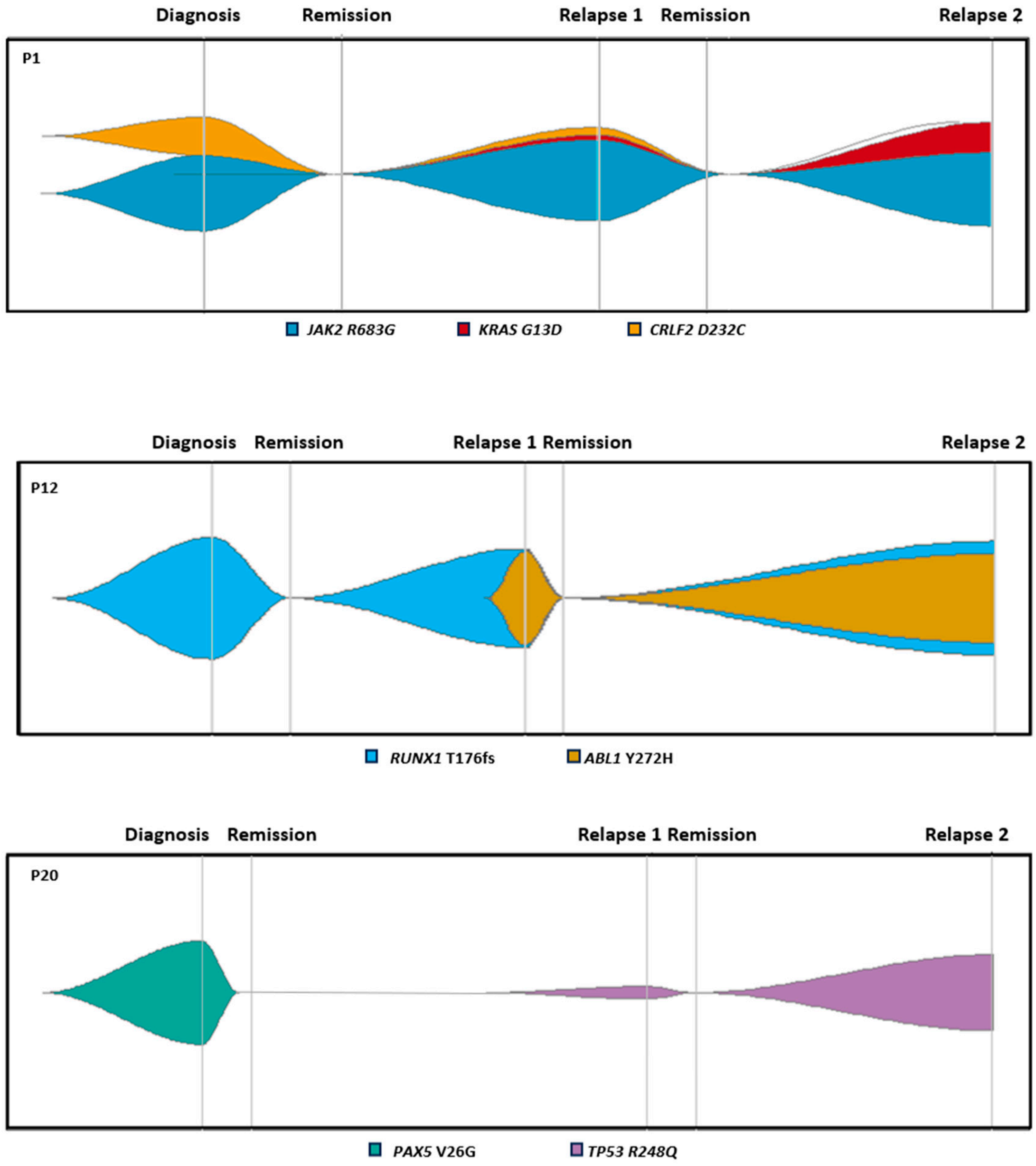

Supplement: Supplementary file 1 [file cancers-16-04200-s001.zip › Supplementary materials Cancers.pdf]
